# Chimeric anti-HLA antibody receptor engineered human regulatory T cells suppress alloantigen-specific B cells from pre-sensitized transplant recipients

**DOI:** 10.3389/fimmu.2025.1601385

**Published:** 2025-08-15

**Authors:** Jaime Valentín-Quiroga, Alejandro Zarauza-Santoveña, Eduardo López-Collazo, Leonardo M. R. Ferreira

**Affiliations:** ^1^ Department of Pharmacology and Immunology, Medical University of South Carolina, Charleston, SC, United States; ^2^ Hollings Cancer Center, Medical University of South Carolina, Charleston, SC, United States; ^3^ The Innate Immune Response Group, Hospital La Paz Institute for Health Research (IdiPAZ), University Hospital La Paz, Madrid, Spain; ^4^ Paediatric Nephrology Unit, University Hospital La Paz, Madrid, Spain

**Keywords:** HLA sensitization, regulatory T cells, B cells, antibody production, transplantation, engineered immune receptors, human immunology, transplant rejection

## Abstract

Organ transplantation is a lifesaving procedure, with 50,000 transplants happening every year in the United States. However, many patients harbor antibodies and B cells directed against allogeneic human leukocyte antigen (HLA) molecules, notably HLA-A2, greatly decreasing their likelihood of receiving a compatible organ. Moreover, antibody-mediated rejection is a significant contributor to chronic transplant rejection. Current strategies to desensitize patients non-specifically target circulating antibodies and B cells, resulting in poor efficacy and complications. Regulatory T cells (Tregs) are immune cells dedicated to suppressing specific immune responses by interacting with both innate and adaptive immune cells. Here, we genetically modified human Tregs with a chimeric anti-HLA antibody receptor (CHAR) consisting of an extracellular HLA-A2 protein fused to a CD28-CD3zeta intracellular signaling domain, driving Treg activation upon recognition of anti-HLA-A2 antibodies on the surface of alloreactive B cells. We find that HLA-A2 CHAR Tregs get activated specifically by anti-HLA-A2 antibody-producing cells. Of note, HLA-A2 CHAR activation does not negatively affect Treg stability, as measured by expression of the Treg lineage transcription factors FOXP3 and HELIOS. Interestingly, HLA-A2 CHAR Tregs are not cytotoxic towards anti-HLA-A2 antibody-producing cells, unlike HLA-A2 CHAR modified conventional CD4^+^ T cells. Importantly, HLA-A2 CHAR Tregs recognize and significantly suppress high affinity IgG antibody production by B cells from HLA-A2 sensitized patients. Altogether, our results provide proof-of-concept of a new strategy to specifically inhibit alloreactive B cells to desensitize transplant recipients.

## Introduction

Organ transplantation represents a pivotal advancement in modern medicine, offering a lifeline to thousands of patients suffering from end-stage organ failure. Renal transplant is the most common organ transplant worldwide, according to the latest Global Observatory on Donation and Transplantation (GDOT) report (1, 2).

However, several hurdles remain in the field of organ transplantation. A significant barrier to successful transplantation is immune rejection of the donor organ by the recipient’s immune system (3). The current standard of care in organ transplant patients involves lifetime multimodal immunosuppressive drug therapy. Broad suppression of the immune suppression by these drugs results in multiple toxicities, including viral infections, nephrotoxicity, neurotoxicity, hyperglycemia, and cancer development (4–8). These side effects are especially pernicious in pediatric transplant recipients, who can suffer growth delays, cognitive impairments, and compounded cancer risk due to continued exposure to steroids and neurotoxic drugs during a critical developmental period (9–12).

Strikingly, 20% of first-time organ recipients and up to 75% of second-time recipients harbor antibodies and B cells directed against allogeneic human leukocyte antigen (HLA) molecules (13–15). HLA-A2 is a very common HLA allele group; 25% of renal transplant recipients in Europe and the United States receive an HLA-A2 mismatched renal transplant (16, 17). These HLA sensitized patients face an uphill battle in securing compatible grafts: as the risk of antibody-mediated rejection escalates, the pool of eligible donor organs narrows (18), creating the need for higher doses of immunosuppressants (19) and contributing to chronic rejection (20).

Current desensitization protocols to mitigate the effects of these alloreactive antibodies lack specificity, targeting total circulating antibodies or B cells, resulting in poor efficacy and unintended complications (19). It is thus imperative to develop treatments that specifically target the recipient’s alloreactive B cells. Using a cellular approach instead of a broad pharmacological approach could increase efficacy and help prevent non-specific side effects.

Regulatory T cells (Tregs), a subset of CD4^+^ T cells integral to the maintenance of immune tolerance, play a pivotal role in modulating immune responses against self and non-self antigens. These specialized immune cells exert their suppressive functions through various mechanisms, including the inhibition of effector T cell activation and the modulation of B cell responses (21–23). Due to these tolerogenic properties, several ongoing trials focus on using Tregs as cellular therapeutics to replace or diminish the dose of immunosuppressive drugs needed to prevent organ transplant rejection (22, 24–26).

One strategy to reverse HLA pre-sensitization is thus to engineer Tregs to recognize donor HLA specific B cells and suppress their function, leading to immune tolerance specifically to the target allogeneic HLA molecule without affecting immunity to other antigens. Such an approach can help more patients become eligible for organ transplants and reduce the need for broad immunosuppressive regimens in transplant recipients, as well as potentially quell chronic transplant rejection. To accomplish this, we genetically modified human Tregs with a chimeric anti-HLA antibody receptor (CHAR) consisting of an extracellular HLA-A2 protein fused to a CD28-CD3zeta intracellular signaling domain (27, 28), driving Treg activation upon recognition of anti-HLA-A2 antibodies on the surface of alloreactive B cells, and assessed HLA-A2 CHAR Treg phenotype and suppressive function towards HLA-A2 sensitized patient B cells.

## Materials and methods

### Molecular biology

The HLA-A2-CHAR-2A-NGRFt lentiviral plasmid was synthesized by VectorBuilder (Chicago, IL). The construct contained an MND promoter driving the expression of HLA-A2 fused to a CD8 hinge (H), CD28 transmembrane domain (TM), and a CD28−CD3zeta tandem intracellular signaling domain, followed by a T2A sequence and a truncated nerve growth factor receptor (NGFRt) as a reporter gene, similar to constructs reported in (27, 28). The HLA-A2 sequence used was the HLA-A*02010101 coding sequence (CDS) from the IPD-IMGT/HLA database, previously validated for cell surface expression in K562 cells and activation of anti-HLA-A2 CAR Tregs (29). CHAR Lentivirus particles were produced by VectorBuilder and shipped to the laboratory, where they were stored in aliquots at -80°C until use.

### Treg sorting, transduction, and expansion

Human Treg isolation, lentiviral transduction, and *ex vivo* expansion was carried out as previously described (30). Human peripheral blood leukopaks from de-identified HLA-A2 negative healthy donors were purchased from STEMCELL Technologies (Vancouver, Canada). CD4^+^ T cells were enriched using the EasySep Human CD4^+^ T Cell Isolation Kit (STEMCELL Technologies), following manufacturer’s instructions. Enriched CD4^+^ T cells were then stained for CD4, CD25, and CD127, and CD4^+^CD25^hi^CD127^low^ regulatory T cells (Tregs), previously shown to be *bona fide* Tregs (31, 32), and CD4^+^CD25^low^CD127^hi^ effector T (Teff) cells were purified by fluorescence-assisted cell sorting (FACS) using a BD FACS Aria II Cell Sorter (Beckton Dickinson, Franklin Lakes, NJ). Post-sort analyses confirmed greater than 99% purity. Tregs were activated in complete medium (RPMI10), comprising RPMI 1640 medium supplemented with 10% fetal bovine serum (FBS), glutamax, penicillin-streptomycin, HEPES, non-essential amino acids (NEAA), and sodium pyruvate (all from Gibco, ThermoFisher Scientific) with anti-CD3/CD28 beads (Gibco, ThermoFisher Scientific) at a 1:1 ratio and 1,000 international units (IU) per ml of recombinant human IL-2 (Peprotech, ThermoFisher Scientific) at 10^6^ cells/ml in 24-wells (33). 48 hours after activation, Tregs were transduced with CHAR lentivirus at a multiplicity of infection (MOI) of 3 (3 particles per cell) in the presence of IL-2. After adding the lentivirus, Tregs cells were centrifuged at 1,000 g at 32°C for 1 hour. Following transduction, Tregs were maintained and expanded in RPMI10, with fresh medium and IL-2 being given every two days. Tregs received 1,000 IU/ml IL-2 and CD4^+^ Teff cells received 100 IU/ml IL-2 for 9–12 days. CHAR transduction efficiency was evaluated by flow cytometry based on HLA-A2 and NGFRt reporter surface expression.

### Activation assay

Untransduced (UT) or CHAR Tregs were co-cultured with irradiated (4,000 rad) B-cell hybridoma cell lines (kind gift from Instituto Salud Carlos III, Madrid, Spain) specific for HLA-A2 (PA2.1) or HLA-DR (IVA12) at a 1:1 ratio in RPMI10 medium supplemented with 1,000 IU/ml IL-2 in round-bottom 96-wells. CHAR Tregs alone and UT Tregs alone or co-incubated with IVA12 or PA2.1 cells served as a negative controls. Surface expression of the T cell activation markers CD69, CD71, and CD25 in UT or CHAR^+^ Tregs was assessed 48 hours later by flow cytometry. Parallel co-cultures were kept for 9 days to assess selective enrichment in NGFRt^+^ Tregs as an additional metric of activation.

### Treg stability assessment

CHAR Tregs were co-cultured with irradiated PA2.1 or IVA12 B-cell hybridoma cell lines at a 1:1 ratio in RPMI10 medium supplemented with 1,000 IU/ml IL-2 in round-bottom 96-wells. UT and CHAR Tregs alone served as controls. Cells were expanded and expression of the Treg lineage transcription factors FOXP3 and HELIOS was assessed 9 days post activation by intracellular staining using the FOXP3/Transcription Factor Staining Buffer Set (eBioscience, ThermoFisher Scientific), according to manufacturer’s instructions. Teff cells were stained for FOXP3 and HELIOS as negative controls.

### Cytotoxicity assay

CHAR Tregs, CHAR Teff cells or their UT counterparts were co-incubated with PA2.1 cells at a 1:1 ratio for 24 hours in round-bottom 96-wells. 50 μl supernatant was then carefully removed and target cell killing assessed using the CyQUANT Cytotoxicity Lactate Dehydrogenase (LDH) Release Assay kit (Thermofisher Scientific) as per manufacturer’s instructions (34).

### Patient peripheral blood mononuclear cell isolation

HLA-A2 sensitized patient whole blood was collected in EDTA tubes. Peripheral blood mononuclear cells (PBMCs) were immediately isolated using a Ficoll-Paque gradient and frozen in liquid nitrogen at University Hospital La Paz, Madrid, Spain. De-identified frozen patient PBMCs were shipped to the Medical University of South Carolina.

### Allogeneic B cell stimulation

Peripheral blood mononuclear cells (PBMCs) from de-identified HLA-A2 pre-sensitized patients (Paediatric and Adult Nephrology Unit, La Paz University Hospital, Madrid, Spain) were thawed in RPMI10, counted, and the frequency of B cells (CD19^+^CD20^+^ cells) was determined by spectral flow cytometry. On the same day, PBMCs were incubated with 100 IU/ml IL-2, 100 IU/ml IL-6 (Peprotech, ThermoFisher Scientific) (35), and irradiated (4,000 rad) HLA2-expressing K562 cells (a kind gift from Jack Strominger, Harvard University) at a ratio of 1 B cell: 10 irradiated HLA-A2-K562 cells with or without HLA-A2 CHAR Tregs at a ratio of 1 CHAR Treg: 1 B cell in 12-wells for up to 5 days. PBMCs from pre-sensitized patients with IL-2 and IL-6 alone, as well as PBMCs from a de-identified healthy donor (STEMCELL Technologies) subjected to all 3 conditions, were kept as negative controls.

### IgG antibody production assay

Three conditions were set up with HLA-A2 pre-sensitized patient PBMCs and healthy donor PBMCs: PBMCs alone, PBMCs with irradiated HLA2-K562 cells, and PBMCs with irradiated HLA2-K562 cells and HLA-A2 CHAR Tregs, as described above. All cultures received 100 IU/ml IL-2 and 100 IU/ml IL-6. After 48h or 5 days of culture, 250 μl supernatant was collected from each allogeneic B cell stimulation condition and diluted 1:2 with PBS. Human IgG solid-phase sandwich enzyme-linked immunosorbent assay (ELISA) (Thermofisher Scientific) was performed as per manufacturer’s instructions.

### Spectral flow cytometry

Spectral flow cytometry data was acquired in a 3-laser Cytek Northern Lights spectral flow cytometer (Cytek Biosciences, Fremont, CA). Spectroflow 3.2.1 (Cytek Biosciences) and FlowJo v10.9 software (BD Life Sciences, Franklin Lakes, NJ) were used for flow cytometry data analysis. Data were manually pre-gated to remove cell aggregates, dead cells, debris, and then sub-sampled to include 10,000 live singlets from each sample. Uniform Manifold Approximation and Projection (UMAP) analysis was performed to visualize the different sub-populations in groups (36). UMAP settings were as follows: all files used, all compensated fluorescent parameters were used besides Live/Dead,Neighbors =15, Minimum Distance = 0.5, Components = 2, Metric = Euclidean. Antibodies used for spectral flow cytometry in this study can be found in **Supplementary Table 1**.

### Ethics approval statement

The studies involving human participants were reviewed and approved by the Local Committee for Research Ethics at the University Hospital La Paz, Madrid, Spain (PI-3969, principal investigator: ELC). Written informed consent to participate in this study was provided by the participants. Samples were collected by AZS and processed by JVQ.

## Results

**Figure 1:**
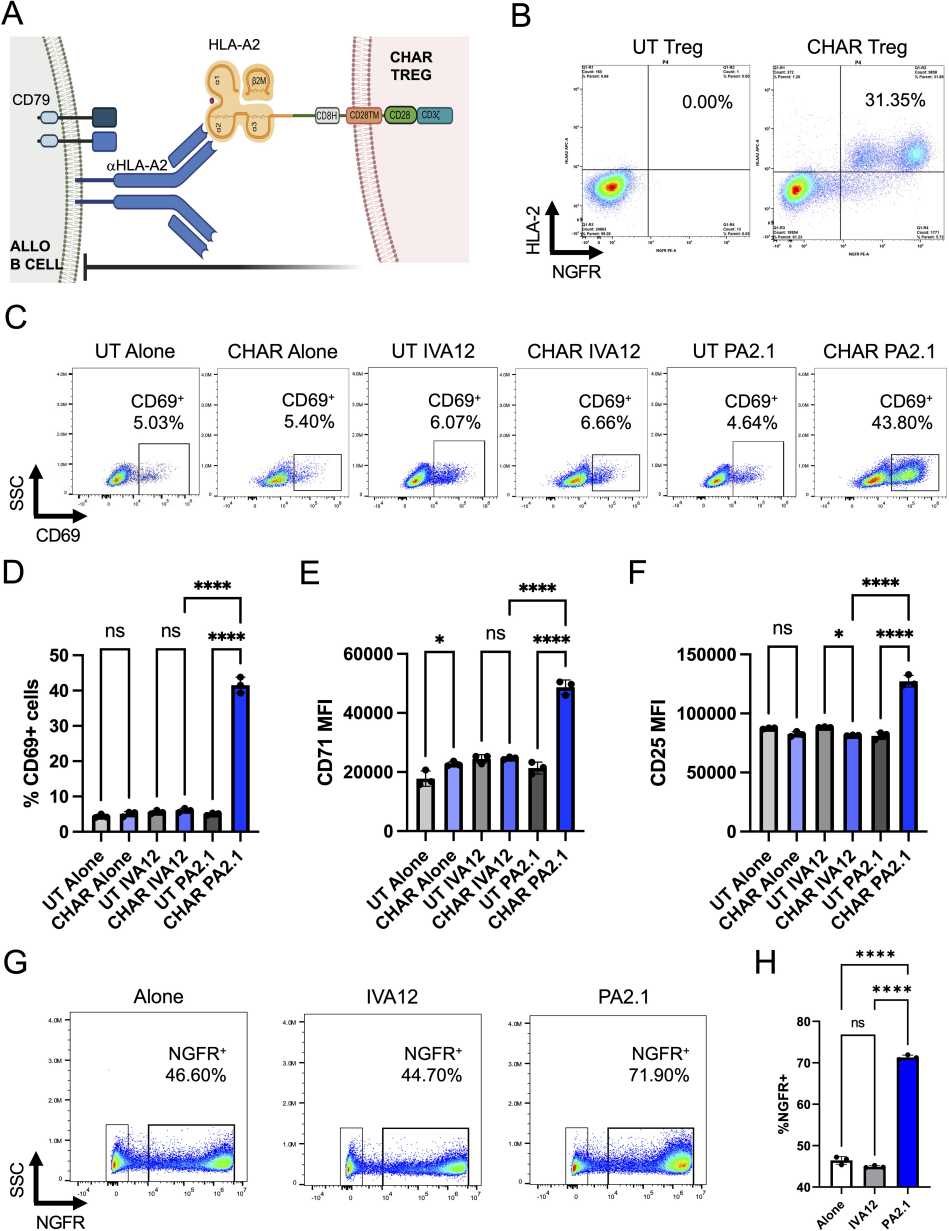
HLA-A2 CHAR Tregs are activated specifically by anti-HLA-A2 antibody producing cells. **(A)** Schematic representation of chimeric anti-human leukocyte antigen (HLA) antibody receptor (CHAR) featuring a CD28-CD3zeta signaling domain, expressed on the surface of a human regulatory T cell (Treg), binding an anti-HLA-A2 antibody on the surface of an allogeneic B cell from an HLA-A2 pre-sensitized patient. The B cell receptor (BCR) on the surface of the B cell comprises a surface-bound antibody and the signaling heterodimer CD79A and CD79B. Upon engagement, the HLA-A2 CHAR Treg suppresses anti-HLA-A2 expressing B cell function. **(B)** Cell surface expression of HLA-A2 CHAR construct in lentivirus transduced Tregs, as assessed by co-expression of HLA-A2 and a reporter gene, truncated nerve growth factor receptor (NGFRt), linked to the CHAR gene by a 2A peptide. UT, untransduced. **(C)** CHAR Treg activation upon 48-hour co-incubation with irradiated PA2.1 (anti-HLA-A2), but not alone or with IVA12 (anti-HLA-DR, DP, DQ) hybridoma cells, as assessed by surface expression of CD69. **(D)** Frequency of CD69-expressing cells among UT or CHAR Tregs alone or co-incubated with PA2.1 or IVA12 cells for 48h. **(E)** Expression levels (MFI, median fluorescence intensity) of CD71 on UT or CHAR Tregs alone or co-incubated with PA2.1 or IVA12 cells for 48h. **(F)** Expression levels (MFI) of CD25 on UT or CHAR Tregs alone or co-incubated with PA2.1 or IVA12 cells for 48h. **(G)** Enrichment of CHAR-expressing Tregs upon 9-day co-incubation with irradiated PA2.1 cells, but not alone or with IVA12 cells, as assessed by surface expression of NGFRt reporter. **(H)** Frequency of NGFRt-expressing Tregs (CHAR Tregs) alone or co-incubated with PA2.1 or IVA12 cells for 9 days. For **(D–F, H)**, bars represent mean and standard deviation (n = 3 technical replicates, one blood donor representative of two). Data were analyzed by one-way ANOVA with multiple comparisons. ns, not significant; *p < 0.05, **p < 0.01, ***p < 0.001, and ****p < 0.0001.

Aiming to test the concept of regulatory T cell (Tregs) engineered to specifically recognize and inhibit alloreactive B cells responsible for human leukocyte antigen (HLA) pre-sensitization in patients, we constructed a chimeric anti-HLA antibody receptor (CHAR) specific for anti-HLA-A2 B cells by fusing an HLA-A2 molecule to a CD8 hinge, a CD28 transmembrane domain, and a CD28-CD3zeta intracellular signaling domain (27, 28) (**Figure 1A**). We then sorted CD4^+^CD25^hi^CD127^low^ Tregs (31, 32) from human peripheral blood collected from HLA-A2 negative donors, activated them with anti-CD3/CD28 beads and IL-2, and transduced them two days later with lentivirus coding for the HLA-A2 CHAR. Flow cytometry analysis of transduced Tregs revealed successful expression of the CHAR construct, as assessed by simultaneous surface expression of HLA-A2 and the truncated nerve growth factor receptor (NGFRt) reporter gene, linked to CHAR gene expression via a 2A peptide (**Figure 1B**).

To evaluate CHAR target recognition, we co-incubated untransduced (UT) or CHAR Tregs with B cell hybridoma cell lines specific for HLA-A2 (PA2.1) or for HLA-DR (IVA12). CHAR Tregs, but not UT Tregs, upregulated surface expression of the well-established Treg activation markers (30, 37) CD69 (**Figures 1C, D**), CD71 (**Figure 1E**), and CD25 (**Figure 1F**) and increased in frequency (**Figures 1G, H**) upon co-incubation with PA2.1 cells, but not with IVA12 cells, indicating CHAR Treg reactivity specifically to HLA-A2 antibody-producing cells.

**Figure 2:**
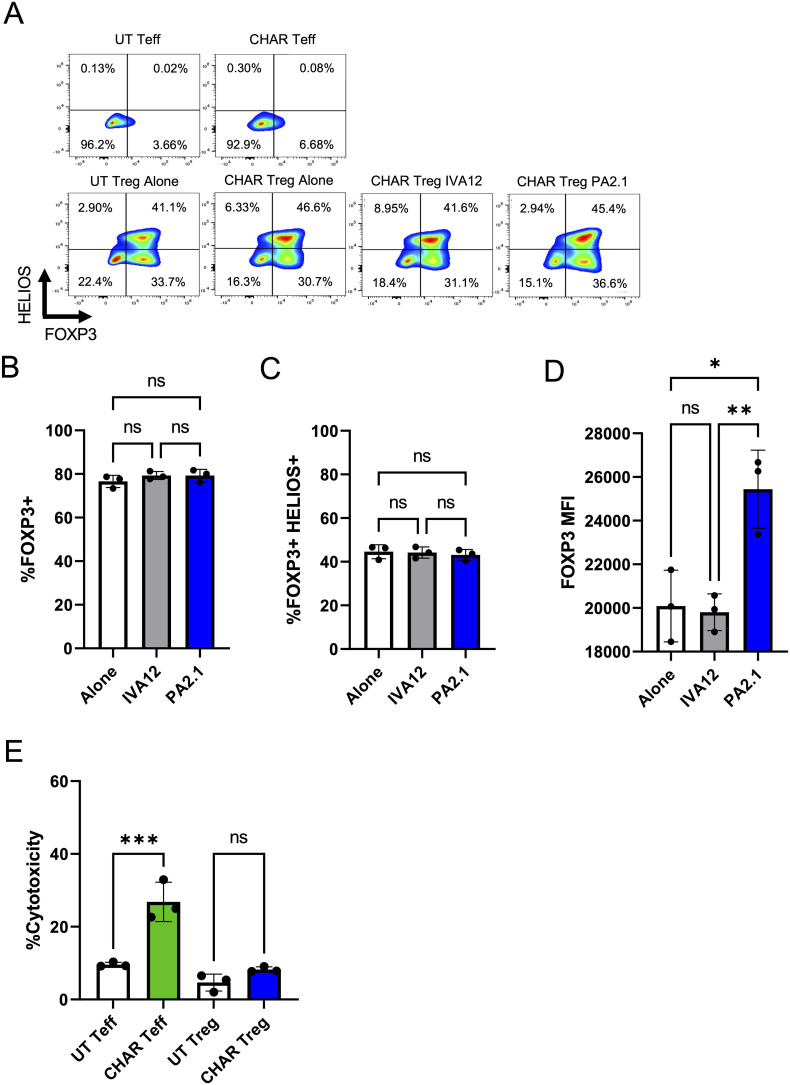
HLA-A2 CHAR Tregs remain stable and are not cytotoxic upon activation. **(A)** Representative flow cytometry analysis of CHAR Treg FOXP3 and HELIOS expression after 9 days of co-culture with irradiated PA2.1 (anti-HLA-A2) and IVA12 (anti-HLA-DR, DP, DQ) hybridoma cells. Untransduced (UT) T effector (Teff) cells and CHAR Teff cells were used as negative controls. **(B)** Frequency of FOXP3^+^ CHAR Tregs alone or co-cultured with PA2.1 and IVA12 hybridoma cells for 9 days. **(C)** FOXP3 expression (mean fluorescence intensity - MFI) in CHAR Tregs alone or co-cultured with PA2.1 and IVA12 hybridoma cells for 9 days. **(D)** Frequency of FOXP3+HELIOS+ CHAR Tregs alone or co-cultured with irradiated PA2.1 and IVA12 hybridoma cells for 9 days. **(E)** Cytotoxicity of CHAR Tregs and CHAR Teff cells towards PA2.1, as measured by lactate dehydrogenase (LDH) release after 24-hour co-incubation at a 1:1 ratio. Bars in **(B–E)** represent mean and standard deviation (n = 3 technical replicates, one blood donor representative of two). Data were analyzed by one-way ANOVA with multiple comparisons. ns, not significant; *p < 0.05, **p < 0.01, ***p < 0.001, and ****p < 0.0001.

Next, we sought to confirm that activation via the CHAR did not compromise Treg identity stability, measured by the expression levels of the Treg lineage transcription factors FOXP3 and HELIOS (38). We found no difference in the frequency of FOXP3 or HELIOS expressing cells between UT or CHAR Tregs co-incubated with either PA2.1 or IVA12 hybridoma cells (**Figure 2A**). Of note, while the frequency of FOXP3^+^ CHAR Tregs (**Figure 2B**) and FOXP3^+^HELIOS^+^ CHAR Tregs (**Figure 2C**) did not change across conditions, FOXP3 levels were higher in CHAR Tregs co-incubated with PA2.1 cells (**Figure 2D**), an additional line of evidence for CHAR Treg activation specifically by anti-HLA-A2 antibody-producing cells (39).

Interestingly, CHAR Tregs were not cytotoxic towards anti-HLA-A2 antibody-producing PA2.1 cells at a 1:1 ratio, unlike CHAR T effector (Teff) cells (**Figure 2E**). While additional donor Tregs need to be tested due to variation in Treg cytotoxic potential in the human population (40), our observations suggest that HLA-A2 CHAR Tregs do not function primarily by eliminating target B cells.

**Figure 3:**
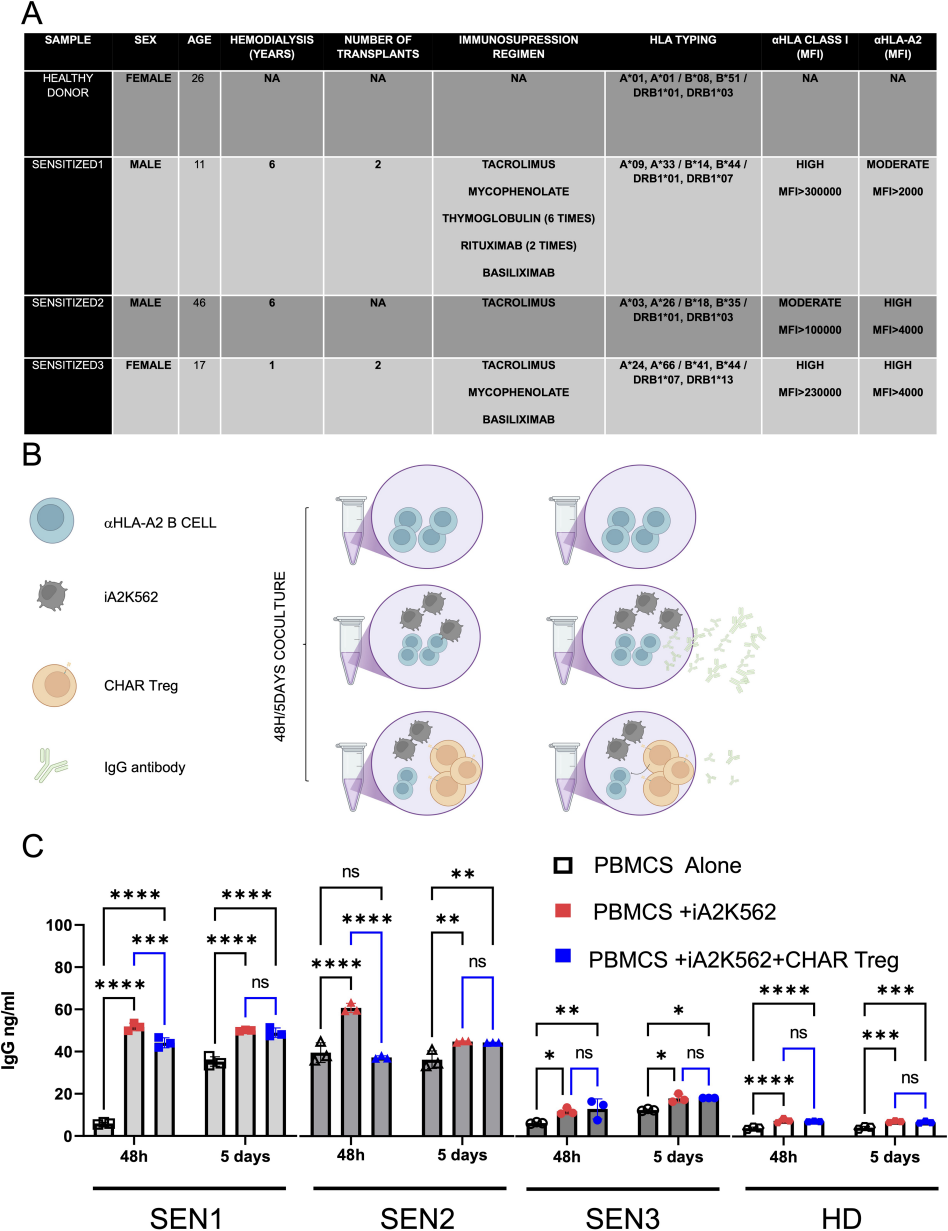
HLA-A2 CHAR Tregs suppress IgG specific production on highly pre sensitized patients. **(A)** HLA-A2 sensitized patient (SEN) and healthy control (HD) demographics and clinical characteristics. **(B)** Experimental design for assessing CHAR Treg function in the presence of HLA-A2 pre-sensitized patient cells. HLA-A2 sensitized donor-derived peripheral blood mononuclear cells (PBMCs) were co-incubated with HLA-A2-expressing K562 cells to induce expansion of anti-HLA-A2 B cells and anti-HLA-A2 IgG antibody production. If CHAR Tregs are added, a decrease in antibody production elicited by exposure to HLA-A2 is expected. **(C)** IgG antibody production 48 hours or 5 days after pre-sensitized patient PBMC co-incubation with HLA-A2-K562 in the presence or absence of CHAR T regs, as assessed by ELISA. n=3 sensitized patients (SEN) and n=1 healthy donor (HD) control). Data were analyzed by one-way ANOVA with multiple comparisons. ns, not significant; *p < 0.05, **p < 0.01, ***p < 0.001, and ****p < 0.0001.

To assess CHAR Treg function, we thawed peripheral blood mononuclear cells (PBMCs) from HLA-A2 pre-sensitized patients (SEN) and a healthy donor (HD) (**Figure 3A**) and co-incubated them with irradiated HLA-A2-expressing K562 cells as a source of HLA-A2 antigen in the presence of IL-2 and IL-6 with or without CHAR Tregs for 2 days or 5 days (**Figure 3B**). In two out of three HLA-A2 pre-sensitized patients tested, CHAR Tregs significantly decreased IgG antibody production by the patient’s cells after 48h of co-incubation (**Figure 3C**), demonstrating the ability of CHAR Tregs to inhibit alloreactive B cells.

**Figure 4:**
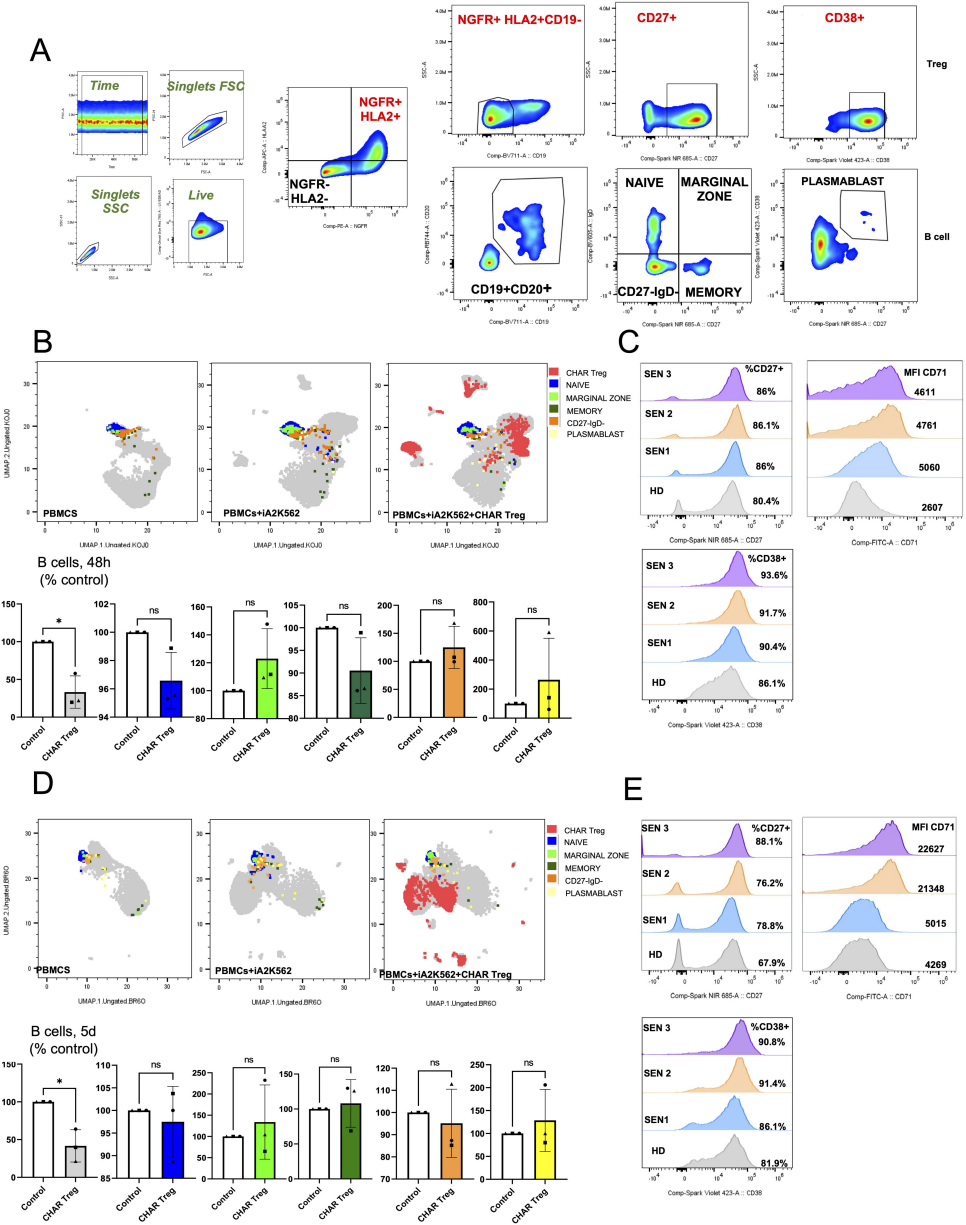
HLA-A2 CHAR Tregs were activated and reduced the frequency of total B cells in pre-sensitized patient peripheral blood samples. **(A)** Flow cytometry gating strategy to identify HLA-A2^+^NGFR^+^CD19^-^ CHAR Tregs, CD19^+^CD20^+^ B cells, and B cell subsets in co-cultures of sensitized patient PBMCs with irradiated HLA-A2-K562 and CHAR Tregs. **(B, D)** Uniform manifold approximation and projection (UMAP) representation of sensitized patient PBMCs with irradiated HLA-A2-K562 (iA2K562) and CHAR Tregs depicting naive, marginal zone, memory and IgD^-^CD27^-^ B cells, as well as plasmablasts, after 48 hours **(B)** and 5 days **(D)** of co-culture. **(C, E)** Frequency of CD27 expression and CD38 expression, as well as CD71 surface expression levels, in CHAR Tregs co-incubated with sensitized patient PBMCs for 48 hours **(C)** and 5 days **(E)**. n=3 sensitized patients (SEN) and n=1 healthy donor (HD) control. Data were analyzed by one-way ANOVA with multiple comparisons. ns, not significant; *p < 0.05, **p < 0.01, ***p < 0.001, and ****p < 0.0001.

In order to gain mechanistic insight into CHAR Treg function, we performed spectral flow cytometric analysis of these co-cultures. We identified NGFR^+^HLA-A2^+^CD19^-^ CHAR Tregs and NGFR^-^HLA-A2^-^CD19^+^CD20^+^ patient B cells. Expression of CD27 (Treg suppressive function marker (41, 42)), CD71 (Treg activation marker (29, 30, 37)), and CD38 (Treg activation and suppressive function marker (42, 43)) was assessed in CHAR Tregs. B cells were subsetted into naïve B cells (CD27^-^IgD^+^), marginal zone B cells (CD27^+^IgD^+^), memory B cells (CD27^+^IgD^-^), CD27^-^IgD^-^ double-negative B cells, and CD27^+^CD38^+^ plasmablasts (**Figure 4A**). Uniform Manifold Approximation and Projection (UMAP) visualization of total live cells in the different co-culture conditions across all three pre-sensitized patients at 48 hours (**Figure 4B**) and 5 days (**Figure 4D**) illustrates that B cells constitute a small and relatively uniform fraction of the patients’ PBMCs (colored in blue, light green, dark green, orange, and yellow according to B cell subset) and that CHAR Tregs (colored in red) form distinct clusters, potentially reflecting differences in activation status. Focusing on the B cell fraction, we found that CHAR Tregs significantly reduced the frequency of total B cells in all three pre-sensitized patients’ PBMCs at 48 hours (**Figure 4B**) and at 5 days (**Figures 4D, E**) of co-culture. Of note, the frequencies of any of the individual B cell subsets measured (naïve, memory, marginal zone, and CD27^-^IgD^-^ B cells, as well as plasmablasts) were not significantly altered by CHAR Tregs at either time point (**Figures 4B, D**), suggesting that all B cell subsets were equally suppressed by CHAR Tregs. With regards to the CHAR Tregs, we observed that the majority of CHAR Tregs expressed high levels of CD27 and CD38, markers associated with Treg suppressive function (41–43) after 48h (**Figure 4C**) and 5 days (**Figure 4E**) of co-culture. There was a trend where a larger fraction of CHAR Tregs expressed CD27 and CD38 when co-incubated with SEN PBMCs (86% CD27^+^ and 90-94% CD38^+^ at 48 hours, 76-88% CD27^+^ and 86-91% CD38^+^ at 5 days) than with HD PBMCs (80% CD27^+^ and 86% CD38^+^ at 48 hours, 68% CD27^+^ and 82% CD38^+^ at 5 days) (**Figures 4C, E**). Moreover, CHAR Tregs were activated, as assessed by CD71 upregulation (29, 30, 37), in the presence of all three SEN PBMCs, but not HD PBMCs after 48 hours (**Figure 4C**) and 5 days (**Figure 4E**) of co-culture.

## Discussion

The field of immunotherapy has witnessed remarkable advancements in recent years, particularly concerning the engineering of T cells to target specific cells and immune responses. Previous studies have demonstrated the potential of conventional T cells to recognize and eliminate B cells. In the clinic, total B cells are being eliminated using CD19 chimeric antigen receptor (CAR) T cells in patients with systemic lupus erythematosus and other autoimmune disorders, leading to disease remission (44, 45). At the pre-clinical stage, self-reactive antigen-specific B cells have been eliminated through chimeric autoantibody receptor T cells (CAAR T cells) in the setting of pemphigus vulgaris (46) and alloreactive donor HLA-specific B cells have been targeted using CHAR T cells (27, 28).

Our study introduces a novel approach by engineering regulatory T cells (Tregs), immune cells dedicated to inhibiting immune responses to maintain immune homeostasis (21–23), to recognize and suppress B cells specific for an HLA molecule with a CHAR. This innovative strategy not only enhances the specificity of Treg-mediated suppression of alloreactive B cells but also opens new avenues for therapeutic intervention in transplant immunology. Tregs, like all CD4^+^ T cells, possess T cell receptors (TCRs) restricted to human leukocyte antigen (HLA) class II molecules, which are expressed in professional antigen presenting cells, including B cells (47). Previous work reported the expansion of rare recipient alloreactive Tregs using donor-derived B cells (48). Yet, antigen-specific Tregs are rare and prone to destabilization upon multiple rounds of activation *ex vivo*, kindling interest in genetic engineering approaches to confer desired antigen specificities to human Tregs (21, 22). Previous work has focused on modifying human Tregs with artificial receptors to confer them specificity towards transplanted tissues, aiming to provide localized protection from immune attack (29, 37, 49–51). Engineering Tregs with CHAR tackles the problem of allogeneic immune rejection from a different, potentially orthogonal and synergistic, angle, providing a targeted mechanism to directly inhibit B cell responses against allogeneic HLA molecules, such as HLA-A2. Unlike engineered conventional T cells, which can induce wide-ranging immune activation and potential tissue damage (52–55), HLA-A2 CHAR Tregs are designed to suppress only B cells producing anti-HLA-A2 antibodies in an anti-inflammatory fashion, thereby minimizing collateral damage to other components of the immune system or any tissues. In line with this concept, we observed that while conventional HLA-A2 CHAR T cells were cytotoxic towards anti-HLA-A2 antibody-producing hybridoma cells, HLA-A2 CHAR Tregs were not (**Figure 2E**). Future studies will manufacture CHAR Tregs from more blood donors to ascertain that CHAR Tregs are not cytotoxic regardless of origin, as variation in the cytotoxic potential of engineered Tregs towards target cells has been observed in patient samples (40), as well as characterize the cytokine secretion of activated CHAR Tregs.

Of note, Tregs are more amenable to be used as allogeneic cell therapies than conventional T cells, which carry the risk of inducing graft-vs-host disease unless they endogenous TCR expression is eliminated (56–58), potentially allowing for the development of off-the-shelf CHAR Treg therapeutics. Experiments using polyclonal UT Tregs will be key to explore this possibility: while UT Tregs were not activated by allogeneic B cell hybridomas *in vitro* (**Figures 1C-F**), mouse studies have shown that polyclonal Tregs are activated by allogeneic DCs and are suppressive *in vivo* (59–61), potentially leading to unwanted suppression beyond specific alloreactive B cell inhibition. Elimination of the endogenous TCR may thus be required for future allogeneic CHAR Treg therapies.

Tregs have been shown to directly inhibit B cells via contact-dependent mechanisms, either by inhibiting B cell proliferation through ligation of PD-L1 and PD-L2 on Tregs with PD-1 on B cells (62), or by killing B cells via the perforin/granzyme B pathway, FASL, or PD-L1 and PD-L2 (62–64). Tregs can also directly inhibit B cells via contact-independent mechanisms, mainly by secreting suppressive cytokines IL-10 and TGF-β, which inhibit B cell proliferation (65, 66). Interestingly, these cytokines also promote B cell differentiation into IL-10- and TGF-β-secreting regulatory B cells (Bregs), which can in turn induce naïve T cell differentiation into Tregs (22, 67, 68). Follow-up studies will profile surface protein markers, cytokine secretion, and the transcriptome of activated CHAR Tregs, coupled with loss-of-function perturbations and immune assays, to dissect the mechanisms by which CHAR Tregs inhibit target alloreactive B cells and further validate the anti-inflammatory characteristics of CHAR Tregs.

Specificity and safety profile are crucial in the context of transplantation, where current desensitization strategies often fall short due to their non-specific nature and associated complications (19). By effectively reducing IgG antibody production by sensitized individuals’ B cells (**Figure 3C**) while maintaining their Treg identity (**Figures 2A–D**) *in vitro*, CHAR Tregs offer a promising therapeutic option for improving transplant outcomes by enhancing graft acceptance and reducing the risk of rejection. Demonstrating CHAR Treg long-term persistence, stability, and function under chronic antigen exposure and inflammatory microenvironments in humanized mouse models (69) will lend additional support to translate CHAR Tregs into the clinic.

Engineering of Tregs to bind specific B cells had been previously demonstrated with FVIII B cell antibody receptor (BAR) Tregs, aimed at inhibiting anti-FVIII antibody production by hemophilic patients treated with recombinant FVIII protein (70). While this work demonstrated the possibility of directing Tregs towards B cells based on B-cell antigen specificity, our findings extend the applicability of B-cell-targeting engineered Tregs to graft-vs-host disease (GvHD), organ transplantation, and beyond, potentially addressing challenges in conditions such as miscarriage, where pregnant women develop antibodies against paternally derived HLA molecules (13, 71, 72). Of note, a limitation common to CAAR, BAR, and CHAR receptors is their likely inability to target IgG plasma cells, terminally differentiated IgG antibody-secreting cells that lose surface BCR expression (73), potentially necessitating additional plasma cell targeting interventions for some patients (74). Additionally, while reported to not be an issue with CAAR receptors (46), it will be important to ascertain that high levels of soluble anti-HLA-A2 antibodies do not engage or block HLA-A2 CHAR receptors.

The novelty of our study lies not only in the engineering of Tregs with CHAR but also in the demonstration of their efficacy with HLA sensitized patients’ cells. The ability of CHAR Tregs to suppress IgG production by B cells from pre-sensitized individuals upon exposure to antigen-expressing cells (**Figure 3C**) is a significant step forward, suggesting that this approach could pave the way for tailored strategies that address individual HLA sensitized patient needs. Polyclonal Tregs have been shown to be safe in phase I and phase II clinical trials (75–77), and human CAR Tregs targeting transplanted tissues have shown efficacy and safety in preclinical studies (37, 78–80) and are being tested in ongoing phase I clinical trials (81). Hence, CHAR Tregs are a good candidate for first-in-human trials, to be used either in pre-sensitized patients with the goal of bringing them to the same baseline as non-sensitized patients and/or as an adjuvant to reduce the doses of immunosuppressive drugs taken by transplant recipients.

Interestingly, CHAR Tregs significantly inhibited IgG production by HLA-A2 antigen-stimulated B cells for only two out of three sensitized patients (**Figure 3C**). Moreover, CHAR Tregs retained high expression of the activation marker CD71 after 5 days of co-culture also only with two out of three sensitized patient PBMCs (**Figure 4E**). It is possible that IgG secretion by HLA-A2 antigen-activated B cells from patient SEN3 was not reduced by CHAR Tregs to a statistically significant extent due to the small magnitude of IgG secretion (**Figure 3C**). Developing an ELISA to quantify anti-HLA-A2 antibody production specifically instead of total IgG may increase the sensitivity to detect differences in antibody production by anti-HLA-A2 B cells in response to HLA-A2 CHAR Tregs. Additionally, measuring IgG classes separately can shed light on the mechanisms of CHAR Treg-mediated suppression. For instance, IL-10 has been shown to induce B cells to secrete IgG1 and IgG3 (82), while IL-10-producing Bregs, which can be induced by Tregs, uniquely secrete IgG4 antibodies (83). Future studies with more HLA-A2 sensitized patient samples and healthy donor control samples are warranted to define what patient characteristics indicate responsiveness to CHAR Treg targeting and dissect the mechanisms by which CHAR Tregs inhibit target B cells. In addition, experiments including conditions with HLA-A2 sensitized patient PBMCs, HLA-A2 antigen, and UT Tregs, as well as conditions with PBMCs from patients sensitized to a different HLA allele (e.g. HLA-A24 (84)), that different HLA allele antigen, and HLA-A2 CHAR Tregs, both expected to display unimpeded IgG production, will aid in the characterization of our CHAR Treg-based approach in future comprehensive studies. Finally, including samples from patients sensitized to other HLA alleles and designing and testing matching CHARs will allow us to expand our CHAR Treg concept to other HLA class I and class II molecules.

In conclusion, we provide compelling proof-of-concept for a novel immunotherapeutic strategy to desensitize transplant recipients with HLA sensitization. Engineering CHAR Tregs represents an advancement in the quest for specific and safe immunotherapies to specifically modulate harmful B-cell responses in sensitized transplant recipients and beyond. As we continue to refine these technologies, the potential for engineered Tregs to transform clinical practice becomes a tantalizing prospect.

## Data Availability

The original contributions presented in the study are included in the article/**Supplementary Material**. Further inquiries can be directed to the corresponding author.
